# Bulk Segregant Analysis Integrated with Transcriptomic and Proteomic Approaches Reveals Candidate Genes Associated with Drought Tolerance in Cotton

**DOI:** 10.3390/biology15201902

**Published:** 2026-10-21

**Authors:** Yousheng Tian, Zengqiang Zhao, Yongmei Dong, Youzhong Li, Hang Yu, Juyun Zheng, Zongming Xie

**Affiliations:** 1 Xinjiang Production and Construction Corps Key Laboratory of Crop Germplasm Enhancement and Biobreeding, Cotton Research Institute of Xinjiang Academy of Agricultural and Reclamation Sciences, Shihezi 832000, China; tianyoushengzi@163.com (Y.T.);; 2 Xinjiang Key Laboratory of Cotton Genetic Improvement and Intelligent Production, Cotton Research Institute of Xinjiang Uygur Autonomous Region Academy of Agricultural Sciences, Urumqi 830091, China

**Keywords:** cotton, drought, BSA-seq, RNA-seq, proteome

## Abstract

Drought is one of the most severe and frequently occurring abiotic stresses that significantly threaten cotton production. In this study, to identify key genes associated with drought resistance in upland cotton, BSA-seq was integrated with RNA-seq (R) and proteomic (P) approaches conducted on an F_2_ population and two parental accessions exhibiting extreme drought resistance: the drought-sensitive accession “2406211” (A) and the drought-resistant accession “2403739” (B). After crossing the two parent plants, the F_2_ generation from these accessions was used to construct pools representing the two extreme drought resistance traits. BSA-seq analysis identified five QTL regions on chromosomes A02 (9.10 Mb–11.10 Mb), A07 (18.20 Mb–20.70 Mb), A09 (39.80 Mb–42.40 Mb), D03 (8.90 Mb–13.80 Mb), and D04 (10.80 Mb–13.40 Mb). RNA-Seq analysis revealed a total of 5844 differentially expressed genes (DEGs) in RAW1 (W1, control condition) versus RAW2 (W2, drought stress), comprising 2499 up-regulated and 3345 down-regulated genes. In RBW1 versus RBW2, 6586 DEGs were detected, with 3286 up-regulated and 3300 down-regulated genes. Proteomic analysis identified 1352 differentially expressed proteins (DEPs) in PAW1 versus PAW2, including 453 up-regulated and 899 down-regulated proteins, and 3440 DEPs in PBW1 versus PBW2, with 1773 up-regulated and 1667 down-regulated proteins. By integrating transcriptomic and proteomic data to analyze genes located within the BSA target regions, we identified 5 DEGs/DEPs, including *GH_D04G0687*, *GH_A02G0703*, *GH_A07G1211*, *GH_A07G1202*, and *GH_D04G0702*, as potential candidate genes within the drought-resistance target intervals. This study expands transcriptomic and proteomic resources and identifies novel gene targets for the genetic improvement and breeding of cotton cultivars.

## Simple Summary

Drought stress severely limits cotton yield. In this study, BSA-seq was combined with RNA-seq and DIA proteomics to analyze an F_2_ segregating population and their upland cotton parents exhibiting contrasting drought tolerance (sensitive accession 2406211 and resistant accession 2403739). Five QTL regions associated with drought resistance were identified on chromosomes A02, A07, A09, D03, and D04 through BSA-seq analysis. Numerous differentially expressed genes (DEGs) and proteins (DEPs) were detected under drought stress via transcriptomic and proteomic profiling of the two parental accessions. Integrated multi-omics analysis of genes within these BSA QTL intervals revealed five candidate genes (*GH_D04G0687*, *GH_A02G0703*, *GH_A07G1211*, *GH_A07G1202*, *GH_D04G0702*) linked to drought resistance. This study provides comprehensive multi-omics datasets and promising gene candidates to facilitate molecular breeding for drought tolerance in upland cotton.

## 1. Introduction

Among abiotic stresses, drought is the most frequent and devastating threat to agricultural production [1]. Drought stress triggers a complex cascade of events within plants [2], most importantly widespread alterations in gene expression that enable the coordinated activation of physiological, biochemical, and defensive systems [3,4]. The acquisition of drought tolerance depends on the induced expression of specific genes, which are classified into two groups: those encoding functional proteins and those encoding regulatory proteins [5,6]. Overall changes-whether increases or decreases-in the abundance of stress-responsive genes and proteins can be comprehensively captured through transcriptomic and proteomic profiling.

Transcriptomic and proteomic profiling are both crucial for studying cotton’s adaptive responses to water deficit, as they reveal different layers of gene regulation. Transcriptomics provides a comprehensive overview of transcriptional activity under drought stress, while proteomics reveals precise protein abundance [7]. Integrated transcriptomic and proteomic datasets are effective tools for different species to explore essential genes, proteins, and signaling pathways associated with abiotic stress responses [8,9,10,11,12,13,14]. Omics datasets facilitate global profiling of stress-adaptive responses, greatly advancing our understanding of the complex molecular mechanisms governing stress resistance. 

The method of combining next-generation sequencing (NGS) with bulked segregant analysis (BSA), abbreviated as BSA-seq [15], enables rapid mapping of quantitative trait loci (QTL) and identification of candidate genes controlling extreme phenotypes by constructing mixed pools [16]. This approach has been increasingly applied across various species to precisely define QTLs associated with specific traits. Using BSA-seq, Zheng et al. [15] identified a candidate region on chromosome 9 containing 778 genes linked to visual stay-green in maize. Shen et al. [17] discovered two major QTLs for grain size on chromosomes 1 and 3. Zhou et al. [18] detected three QTLs (*qAT-A07-1*, *qAT-A07-2*, and *qAT-A09-1*) significantly associated with relative elongation of the taproot, screening 656 candidate genes. Geng et al. [19] identified five major genomic regions on chromosomes A06 (43.88–44.63 Mb), A08 (27.63–27.68 Mb), A09 (55.32–55.46 Mb and 57.33–57.58 Mb), and C07 (29.40–29.60 Mb) as candidate loci for seed weight. Wang et al. [20] found multiple SNP and InDel variation sites on chromosomes 2 and 14 of the tea plant. Su et al. [21] detected a total of 11 candidate intervals on chromosomes 3, 5, 6, 7, 13, and 16 associated with flesh firmness at harvest in apples. The integration of BSA-seq with transcriptomics and proteomics has accelerated the identification of candidate genes for complex traits. Several studies have successfully identified novel genes using this multi-omics approach [22,23,24,25,26]. As global water scarcity becomes an increasingly urgent issue, enhancing crop drought tolerance through multi-omics strategies represents a crucial advancement in sustainable agriculture [27]. 

The drought-tolerant accession 2403739 and drought-sensitive accession 2406211 are advanced cotton breeding lines developed and maintained by our research group. In a previous study, 122 cotton germplasm accessions were evaluated for drought tolerance at the seedling stage under pot conditions. Drought tolerance was comprehensively assessed using a comprehensive drought resistance measurement value (D value) calculated from multiple phenotypic traits. Based on the D values, accessions 2403739 and 2406211 were identified as representative drought-tolerant and drought-sensitive materials, respectively, and were therefore selected as the parents for the present study [28]. In this study, BSA-seq combined with transcriptomic and proteomic analyses was conducted on these two cotton accessions and their offspring to identify candidate genes related to drought resistance. Our findings provide a foundational resource for functional genomics, facilitating the identification of candidate genes for molecular breeding and genetic engineering aimed at improving crop stress resilience.

## 2. Materials and Methods

### 2.1. Development of Mapping Population

The drought-resistant accession 2403739 (B) was used as the female parent and crossed with the drought-sensitive accession 2406211 (A) for an inheritance study of the mapping population. After receiving pollen from the male parent, the hybrid seeds collected from the female parent are referred to as “F_1_ seeds.” The plants grown from the germination of these F_1_ seeds are called “F_1_ plants”. The self-pollinated seeds collected from an individual F_1_ plant correspond to the F_2_ family. The F_2_ family was used for BSA-seq analysis. The F_1_ and F_2_ plants of the population were planted in the field from 2024 to 2025 at the experimental station of the Xinjiang Academy of Agricultural Reclamation Sciences, Xinjiang, China. The F_2_ population was sampled and harvested individually by plant. Samples were snap-frozen in liquid nitrogen and stored at −80 °C until use. The seeds collected from each F_2_ plant are referred to as “F_2:3_ seeds”. The plants grown from the germination of these “F_2:3_ seeds” are called “F_2:3_ plants”. The F_2:3_ plants were planted in pots in 2026 in the artificial climate chamber at the Xinjiang Academy of Agricultural Reclamation Sciences. When the F_2:3_ population reached the two-true-leaf stage, they were subjected to drought stress treatment. The drought resistance of the F_2:3_ lines was comprehensively evaluated by measuring indicators such as plant height, fresh weight of the above-ground parts, and dry weight of the above-ground parts. The drought tolerance of each F_2_ genotype was inferred from the phenotypic performance of its corresponding F_2:3_ family, and DNA previously collected from the corresponding F_2_ individuals was used to construct the two extreme bulks for BSA-seq analysis.

### 2.2. Whole Genome Sequencing and BSA-Seq Analysis

DNA was extracted from the leaves using the DNA Extraction Kit (DP305) from TIANGEN Biotech (Beijing, China) following the manufacturer’s instructions. DNA quality was assessed using a BioPhotometer plus spectrophotometer (Eppendorf AG, Hamburg, Germany) and 1% agarose gel electrophoresis. For BSA-seq, two DNA pools were created by mixing equal amounts of DNA from 24 drought-resistant F_2_ individuals (resistance pool) and 24 drought-sensitive F_2_ individuals (sensitive pool). These two DNA pools were sequenced on the BGISEQ-500 platform at the Beina Technology Co., Ltd. (Wuhan, China). After sequencing, clean reads were obtained by removing low-quality and short reads using the SOAPnuke program (v2.1.7) [29] and mapped to the TM-1 reference genome (https://www.cottongen.org/cottongen_downloads/Gossypium_hirsutum/ZJU-TM1_T2T/assembly/TM-1-T2T.fa.gz, accessed on 1 October 2026) using BWA (v2.2.1) with SAMtools (v1.22) [30]. Single-nucleotide polymorphisms (SNPs) and insertions/deletions (InDels) were identified and filtered by removing heterozygous and missing SNPs, as well as InDels, in both the pools and parental lines using GATK (v4.6.2.0) [31]. The SNP-index represents the ratio of reads containing SNPs to the total number of reads [32]. The ΔSNP-index is the difference between the SNP-indices of the two bulks. To identify candidate regions associated with drought resistance, the ΔSNP-index at each locus was calculated by subtracting the SNP-index of the sensitive pool from that of the resistant pool, following the method described previously [30]. To validate the ΔSNP-index results, the Euclidean Distance (ED) algorithm was also applied to identify SNPs and InDels associated with drought resistance, using the equation reported in earlier studies [33,34]. A total of 1000 permutation tests were conducted to determine the 95% and 99% confidence thresholds for screening candidate regions.

### 2.3. Transcriptome and Proteome Sequencing Analysis

Seeds of parent accessions A and B were sown into PVC pots (six seeds per pot) containing 0.5 kg of a mixture of nutrient soil and vermiculite (3:1 *v*/*v*) and placed in a heliogreenhouse. After seedling emergence, the seedlings were thinned to maintain three uniformly growing seedlings per pot. The gravimetric method was used to monitor soil relative water content (SRWC). Drought stress was applied at the two-leaf stage of the cotton seedlings. Soil water content for the control and drought stress treatments was maintained using the gravimetric method as follows: control (W1), 60–65% SRWC; drought stress (W2), 30–35% SRWC. Water was added to each pot daily at approximately 10:00 a.m. After 10 consecutive days of drought treatment, samples were harvested and stored at −80 °C for RNA-seq (R) and proteomic (P) analyses. In this study, RAW1 represents the RNA-seq data of accession A under control conditions; RAW2 represents the RNA-seq data of accession A under drought stress; RBW1 represents the RNA-seq data of accession B under control conditions; RBW2 represents the RNA-seq data of accession B under drought stress; PAW1 represents the proteome of accession A under control conditions; PAW2 represents the proteome of accession A under drought stress; PBW1 represents the proteome of accession B under control conditions; and PBW2 represents the proteome of accession B under drought stress.

Total RNA was extracted using the RNA Plant Plus Reagent Kit (TIANGEN, Beijing, China) according to the manufacturer’s instructions. Three biological replicates were prepared for each sample. The extracted mRNA was enriched using mRNA Capture Beads (Vazyme Biotech Co., Ltd., Nanjing, China). After purification with the beads, the mRNA was fragmented by high-temperature treatment. The fragmented mRNA then served as a template for synthesizing the first strand of cDNA within a reverse transcription enzyme mixture. During the synthesis of the second strand of cDNA, end repair and A-tailing were performed simultaneously. Subsequently, adapters were ligated. PCR library amplification was then conducted, followed by sequencing on the Illumina HiSeq 2500 platform (Illumina Inc., San Diego, CA, USA). The reads were further filtered using fastp (version 0.18.0) [35] to obtain high-quality clean reads. The filtering parameters were as follows: (1) reads containing adapters were removed; (2) reads with more than 10% unknown nucleotides were discarded; and (3) low-quality reads with more than 50% low-quality (Q-value ≤ 20) bases were eliminated. An index of the reference genome was constructed, and the paired-end clean reads were mapped to the Gossypium hirsutum TM-1 reference genome (TM-1_T2T, https://www.cottongen.org/cottongen_downloads/Gossypium_hirsutum/ZJU-TM1_T2T/assembly/TM-1-T2T.fa.gz, accessed on 1 October 2026) using HISAT2 version 2.1.0 [36]. The quantitative analysis utilized the featureCounts tool (version:1.5.0-p3) [37] in the subread software (v2.1.1). DESeq2 was used to identify significant expression differences in genes across the different samples. Differentially expressed genes (DEGs) were identified using thresholds of *p*-value < 0.05 and fold change > 2 or fold change < 0.5 [38].

The total protein from the parent seedlings was extracted using the TCA/acetone method, and the protein concentration was analyzed using a BCA proteomics kit (Thermo Fisher Scientific, Waltham, MA, USA). After enzymatic digestion, the peptides were fractionated by high-pH reverse-phase liquid chromatography and subjected to liquid chromatography coupled with data-independent acquisition mass spectrometry (LC-DIA-MS) analysis. Proteome Discoverer software (Thermo Fisher Scientific, v2.3) was used for database searching and quantitative analysis of the mass spectrometry data. Proteins with a confidence level higher than 99% and detected in at least one treatment were classified as “High” confidence. Peptides with a confidence level above 95% and proteins with a *q*-value < 0.01 were identified as high-reliability proteins. A fold change (FC) of ≥1.2 was used to indicate up-regulated proteins, ≤0.83 for down-regulated proteins, and a *p*-value < 0.05 was set as the threshold for significant differences [39].

Both differentially expressed genes (DEGs) and differentially expressed proteins (DEPs) were subjected to functional annotation and enrichment analysis using the Gene Ontology (GO, http://www.geneontology.org/, accessed on 1 October 2026) and Kyoto Encyclopedia of Genes and Genomes (KEGG, http://www.genome.jp/kegg/, accessed on 1 October 2026) databases.

## 3. Results

### 3.1. Evaluation of Parent Lines and F_2:3_ Population Resistance to Drought

In a previous drought resistance evaluation, cotton accession 2406211 (A) was identified as drought-sensitive, whereas accession 2403739 (B) was drought-tolerant. The two accessions were sown together in a single pot and grown under normal watering conditions until reaching the two-leaf stage. Watering was then withheld until the plants exhibited severe wilting, after which they were rewatered. Five days after rewatering, accession A had withered and died, while accession B resumed growth (Figure 1A).

A population of 122 F_2:3_ individuals derived from the cross A × B was evaluated for drought resistance. The frequency distribution of drought resistance values (D) in this population exhibited continuous variation (Figure 1B), suggesting that drought resistance is likely controlled by multiple genes.

### 3.2. BSA-Seq Analysis

The filtered clean reads of each sample were mapped to the TM-1 reference genome. A total of 69.76, 75.64, 60.62, and 57.94 Gb of raw data were generated for samples 2406211 (A), 2403739 (B), the sensitive pool, and the resistance pool, respectively, representing approximately 28.59×, 30.65×, 25.82×, and 24.68× genome coverage. The Q30 value exceeded 97%, and the GC content ranged from 36.21% to 37.64%. The alignment efficiency of the sequencing was at least 97.41%, with reads perfectly matched to the genome at an efficiency of at least 95.52% (Table 1). A total of 3,373,761 bi-allelic single-nucleotide polymorphisms (SNPs) and 755,820 bi-allelic short insertions and deletions (InDels) were identified. The average densities of SNP and short InDel markers were approximately 1.57 SNPs/kb and 0.37 InDels/kb, respectively (Table S1).

To identify the genomic regions associated with drought resistance, the ΔSNP-index method was used to calculate allele segregation of SNPs and InDels between two extreme DNA pools. A total of five QTL regions were identified at a 95% association threshold (Figure 2). These QTLs were mapped to chromosomes A02 (9.10 Mb to 11.10 Mb), A07 (18.20 Mb to 20.70 Mb), A09 (39.80 Mb to 42.40 Mb), D03 (8.90 Mb to 13.80 Mb), and D04 (10.80 Mb to 13.40 Mb) (Figure 2, Table 2). At a more stringent 99% association threshold, one QTL region was detected on chromosome D03 (8.9 Mb to 12.8 Mb) (Figure 2).

### 3.3. Transcriptome Sequencing Data Analysis

To elucidate the molecular mechanisms underlying the differences in drought tolerance between resistant and sensitive cotton lines, twelve cDNA libraries were sequenced. After filtering out all low-quality sequences and adapters, between 6.44 Gb and 6.48 Gb of clean data were obtained for each cDNA library. The Q30 ratio was above 97.72% (Table S2). More than 93.80% of clean reads were successfully mapped to the reference genome for further analysis (Table S3). The correlation coefficient between each repetition is high, whereas the Pearson correlation coefficient between each treatment is low (Figure S1). Principal component analysis (PCA) revealed that replicates clustered together, with significant differences observed between the groups (Figure S2).

Under control conditions, there were 2334 DEGs between accession A and accession B, of which 842 were up-regulated and 1492 were down-regulated. Under drought stress, accession A exhibited 2499 up-regulated DEGs and 3345 down-regulated DEGs, whereas accession B showed 3286 up-regulated DEGs and 3300 down-regulated DEGs. Notably, accession B had more DEGs than accession A. Comparing accession A and accession B, 2486 DEGs were identified, of which 864 were up-regulated and 1622 were down-regulated (Figure 3A). There were 469 common DEGs identified between RAW1 versus RBW1 and RAW2 versus RBW2 (Figure 3B), 2695 common DEGs identified between RAW1 versus RAW2 and RBW1 versus RBW2, and 64 common DEGs among these four comparison groups (Figure 3B).

GO enrichment analysis revealed that DEGs between RAW1 and RAW2 were primarily enriched in the biological process categories of cellular process, metabolic process, and single-organism process, among others. In the cellular component category, DEGs were mainly enriched in cell, cell part, membrane, and organelle. In the molecular function category, binding and catalytic activity were predominant (Figure S3A). Similar functions were enriched among DEGs between RBW1 and RBW2; additionally, three other functions—extracellular matrix, metallochaperone activity, and translation regulator activity—were enriched compared with DEGs between RBW1 and RBW2 (Figure S3B).

Meanwhile, KEGG pathway enrichment analysis showed that DEGs between RAW1 and RAW2 were involved in 142 pathways (Table S4). Among these, the top 25 significantly enriched pathways included plant-pathogen interaction (ko04626), MAPK signaling pathway–plant (ko04016), plant hormone signal transduction (ko04075), and starch and sucrose metabolism (ko00500), among others (Figure 3C). Similarly, DEGs between RBW1 and RBW2 participated in 144 pathways (Table S5). The top 25 significantly enriched pathways in this comparison also included plant-pathogen interaction (ko04626), MAPK signaling pathway–plant (ko04016), plant hormone signal transduction (ko04075), biosynthesis of secondary metabolites (ko01110), metabolic pathways (ko01100), among others (Figure 3D). Twelve pathways were enriched in both RAW1 versus RAW2 and RBW1 versus RBW2 comparisons, including plant-pathogen interaction (ko04626), MAPK signaling pathway–plant (ko04016), plant hormone signal transduction (ko04075), starch and sucrose metabolism (ko00500), ascorbate and aldarate metabolism (ko00053), galactose metabolism (ko00052), alpha–linolenic acid metabolism (ko00592), alanine, aspartate and glutamate metabolism (ko00250), amino sugar and nucleotide sugar metabolism (ko00520), sulfur metabolism (ko00920), biosynthesis of secondary metabolites (ko01110), glutathione metabolism (ko00480).

### 3.4. Proteomic Profiling Analyses

To quantitatively compare protein level changes in response to drought stress in cotton, we conducted proteomic profiling using data-independent acquisition (DIA) coupled with mass spectrometry. A total of 89,350 peptides and 10,635 proteins were identified using the DIA-targeted extraction quantification algorithm DIA-NN (v2.0.2), validated with a false discovery rate (FDR) of 0.01. The correlation coefficients among the three replicates of each sample ranged from 0.81 to 1.00. The Pearson correlation coefficients among the three replicates of each sample ranged from 0.81 to 1.00. The correlation coefficient between replicates is higher than that between different samples (Table S6, Figure S4). Principal component analysis showed good clustering of biological replicates, indicating distinct molecular profiles among the experimental groups (Figure S5). Under control conditions, 1376 DEPs were identified between accession A and accession B, of which 441 were up-regulated and 935 were down-regulated. Under drought stress, accession A exhibited 453 up-regulated and 899 down-regulated DEPs, whereas accession B showed 1773 up-regulated and 1667 down-regulated DEPs. A total of 2190 DEPs were identified between accession A and accession B under drought stress, with 1098 up-regulated and 1092 down-regulated (Figure 4A). Additionally, 225 common DEPs were identified between PAW1 versus PBW1 and PAW2 versus PBW2; 414 common DEPs were found between PAW1 versus PAW2 and PBW1 versus PBW2; and 62 common DEPs were shared among all four comparison groups (Figure 4B).

GO enrichment analysis revealed that DEPs between PAW1 and PAW2 were primarily enriched in biological processes such as cellular processes, metabolic processes, and single-organism processes. Regarding cellular components, these DEPs were mainly associated with the cell, cell part, and organelle. In the molecular function category, DEPs were predominantly enriched in binding and catalytic activity (Figure S6A). The DEPs between PBW1 and PBW2 were also enriched in similar functions (Figure S6B).

KEGG pathway enrichment analysis revealed that DEPs between PAW1 and PAW2 were significantly involved in 131 pathways (Table S7). Among these, the top 25 significant pathways included metabolic pathways (ko01100), ribosome (ko03010), and endocytosis (ko04144), among others (Figure 4C). In contrast, DEPs between PBW1 and PBW2 were significantly involved in 139 pathways (Table S8). The top 25 significant pathways included metabolic pathways (ko01100), carbon metabolism (ko01200), photosynthesis (ko00195), and biosynthesis of secondary metabolites (ko01110), among others (Figure 4D). Twelve common pathways including photosynthesis (ko00195), oxidative phosphorylation (ko00190), fatty acid metabolism (ko01212), carbon fixation by calvin cycle (ko00710), porphyrin metabolism (ko00860), fatty acid biosynthesis (ko00061), pyruvate metabolism (ko00620), carbon metabolism (ko01200), biosynthesis of cofactors (ko01240), arginine biosynthesis (ko00220), ribosome (ko03010), and metabolic pathways (ko01100) were enriched in both PAW1 versus PAW2 and PBW1 versus PBW2 comparisons (Figure 4C,D).

### 3.5. Association Analysis of Multi-Omics

To rapidly identify candidate genes associated with drought resistance, we conducted a comprehensive association analysis integrating BSA-seq, RNA-seq, and proteomic data. Based on the RNA-seq results, a total of 13 common DEGs were identified within the BSA candidate intervals by intersecting the RAW1 versus RAW2 and RBW1 versus RBW2 comparisons (Figure 5A). These DEGs exhibited similar expression profiles in both accession A and accession B under drought stress (Figure 5C). The BSA candidate intervals were also independently intersected with RAW1 versus RAW2 and RBW1 versus RBW2 comparisons, yielding other 13 and 16 additional DEGs, respectively. The expression profiles and annotations of these DEGs are presented in Figure 5C and Table S9. Based on proteomic data, only one common DEP was identified within the BSA candidate intervals by intersecting the PAW1 versus PAW2 and PBW1 versus PBW2 comparisons (Figure 5B). Five DEPs were identified by intersecting BSA candidate intervals with the proteomic data from accession A, while seventeen DEPs were identified by intersecting BSA candidate intervals with the proteomic data from accession B (Figure 5B, Table 3 and Table S10). Furthermore, four common DEGs/DEPs were identified through the joint analysis of BSA with transcriptome data and BSA with proteome data (Table S11).

By aligning BSA-seq reads from accession A and accession B to the TM-1 reference genome, we further characterized these DEGs, identifying one gene (*GH_A02G0651*) with frameshift mutations, two genes (*GH_A02G0651* and *GH_D04G0687*) with nonsynonymous mutations, and three genes (*GH_A09G0633*, *GH_D04G0694*, and *GH_D04G0719*) exhibiting upstream mutations (Table S12).

To identify drought-resistant candidate genes, a candidate prioritization scheme that assigns values to organize multiple sources of biological evidence was used to score DEGs/DEPs located within the BSA target regions. DEGs/DEPs significantly upregulated in the transcriptome or proteome of any accession were assigned 2 points; those significantly downregulated received 1 point. No points were given if the difference was not significant. Additionally, a score of 2 was awarded if the gene’s functional annotation was related to drought resistance, as determined by searching the Stress Combinations and their Interactions in Plants (SCIP) database [40] using the gene ID of the homologous gene in Arabidopsis thaliana. A score of 1 was given if a mutation was present in the promoter or exon region. Among the 61 co-located genes, 3 genes received a total score of 6, and 2 genes scored 5 (Table S13). These 5 genes are potential candidates within the target interval.

## 4. Discussion

Among abiotic stresses, drought is the single greatest factor limiting global food production [41]. Therefore, identifying new target genes for improving drought responses in crop species is highly valuable. In field environments, drought often coincides with other abiotic stresses such as high temperature and salinity, further complicating research efforts. The continuously changing and complex stress conditions make it more challenging to identify key drought tolerance genes, proteins, and metabolites [9]. Pot-based systems in artificial climate chambers more closely mimic field conditions by allowing a slower rate of water loss, which facilitates acclimation to stress and avoids the occurrence of additional complex stress factors [42]. This study employs a pot cultivation system to apply drought stress treatment to cotton, enabling precise control over experimental environmental conditions and thereby enhancing experimental reproducibility. By integrating transcriptome, proteome, and BSA sequencing data obtained from this system, it is possible to more accurately elucidate the molecular response characteristics of cotton plants to drought stress.

Transcriptome studies exclusively focus on mRNA levels. However, multiple post-transcriptional regulatory mechanisms exist [43]. Consequently, data obtained from transcriptome studies fail to comprehensively represent all plant responses to drought stress and may not align with findings from proteomic analyses [44]. In this study, 5844 and 6586 DEGs (Figure 3A) were identified by RNA-seq in accession A and accession B, respectively, under drought stress. In contrast, under the same stress, the number of DEPs identified by proteomic analysis decreased significantly, with only 1352 and 3440 DEPs (Figure 4A) detected in accession A and accession B, respectively. Venn analysis revealed 64 common DEGs (Figure 3B) and 62 common DEPs (Figure 4B), but only 2 consistent DEGs/DEPs were found (Table S14). The discrepancy between RNA-seq and proteome results may be due to the pervasive effects of post-transcriptional regulatory mechanisms (e.g., mRNA stability) and post-translational modifications, causing expression patterns at the mRNA and protein levels to not always coincide [45]. Our results are consistent with previous studies, which showed that the overwhelming majority of DEPs were not encoded by the corresponding DEGs, indicating a significant inconsistency between protein abundance and transcription levels [46].

Plant responses to abiotic stress involve the modulation of stress-related genes and proteins, which are either suppressed or induced depending on the specific conditions [47]. KEGG pathway enrichment analysis was used to identify the most significant biochemical metabolism and signal transduction pathways involved in DEGs/DEPs [11]. Wang et al. [48] reported that in okra’s response to drought stress, the main pathways of down-regulated DEPs were “metabolic pathways” and “biosynthesis of secondary metabolites.” We also identified these two pathways in the KEGG analysis of DEGs and DEPs. A previous study found that most proteins with altered levels after drought stress were substantially correlated with defense responses, oxidative stress, detoxification, protein synthesis, and energy and carbon metabolism [49]. In our study, KEGG analysis of DEPs also highlighted enrichment in the “carbon metabolism” pathway. Photosynthesis is one of the key processes affected by drought stress, via decreased CO_2_ diffusion to the chloroplast and metabolic constraints [50,51]. In this study, we detected DEGs/DEPs of accession B significantly involved in the pathways of “photosynthesis” and “photosynthesis–antenna proteins” under drought stress (Figure 3D; Figure 4D). Previous omics studies on plant responses to drought stress have identified pathways such as brassinosteroid biosynthesis [52], metabolic pathways [52], and signal transduction [53] at the mRNA or protein levels, which are consistent with our findings.

We analyzed mutations in DEGs/DEPs using re-sequencing data of BSA-seq. Consistent with previous research [54], the re-sequencing results showed that most SNPs/InDels were located in intergenic regions, while five genes harbored functional mutations. Our study also developed a scoring method to screen candidate genes based on differences in DEGs/DEPs within the BSA interval. Previous studies have highlighted that up-regulated DEGs and more abundant DAPs play important roles in abiotic stress resistance in plants [55]; therefore, we assigned a score of 2 to these DEGs/DEPs. Additionally, DEGs/DEPs annotated as related to drought were also assigned a score of 2. All other cases received a score of 1. As a result, five DEGs/DEPs obtained a total score greater than 5, and their homologous genes in Arabidopsis were all annotated as related to drought resistance. These genes were identified as candidate genes for drought resistance.

Five candidate genes were identified as associated with drought resistance. *GH_D04G0687* was annotated as NAD(P)H-quinone oxidoreductase subunit S. Previous studies have reported that NAD(P)H-quinone oxidoreductase subunits are involved in the response to drought stress [56,57]. *GH_A02G0703* was annotated as plant U-box 29. U-box-containing E3 ligases have undergone significant expansion in the plant kingdom and diversified into plant U-box proteins (PUBs) [58], which play crucial roles in regulating plant hormone signal transduction, stress tolerance, and pathogen resistance [59]. *GH_A07G1211* was annotated as NAC domain-containing protein 61. The plant-specific NAC (NAM, ATAF1/2, and CUC2) proteins constitute a major transcription factor family renowned for their roles in various developmental processes, including senescence, secondary cell wall formation, and responses to biotic and abiotic stresses [60]. *GH_A07G1202* was annotated as an alpha/beta-hydrolase superfamily protein. The alpha/beta hydrolase (ABH) superfamily is a widespread and functionally versatile protein fold that serves catalytic roles in both primary and specialized (secondary) metabolism across all domains of life [61]. *GH_D04G0702* was annotated as ethylene-dependent gravitropism-deficient and yellow-green-like 2, a gene not previously reported in the literature. In contrast, a previous study identified ethylene-dependent gravitropism-deficient and yellow-green-like 1 as a nucleus-encoded, plastid-localized, membrane-associated, ATP-independent metalloprotease site-2 protease [62]. These genes hold potential for developing stress-tolerant cotton varieties through targeted breeding and genetic engineering approaches, including transgenic methods and genome editing.

Several limitations of the present study should be acknowledged. First, drought tolerance was primarily evaluated at the seedling stage, whereas drought responses during flowering and boll development may have a more direct impact on cotton yield. Second, the BSA-seq analysis was based on a single F_2_-derived population of relatively limited size, resulting in relatively broad candidate intervals. Third, transcriptomic and proteomic analyses were conducted using the parental accessions rather than the extreme segregating bulks; therefore, the molecular data provide complementary rather than direct expression evidence within the BSA population. Additionally, the pot-based drought treatment may not fully replicate the complexity of field drought conditions. Finally, the five candidate genes identified through multi-omics integration remain putative, and further validation using larger mapping populations, gene expression assays, genetic transformation, gene editing, or other functional approaches is required to establish their causal roles in cotton drought tolerance.

## 5. Conclusions

This study conducted drought resistance identification on an F_2:3_ population derived from drought-resistant and drought-sensitive parental accessions. Based on the results, 24 F_2_ individual plants were selected separately to construct extreme drought-resistant and drought-sensitive pools. BSA-seq analysis identified five genomic intervals associated with cotton drought resistance. Transcriptome and proteome analyses were employed to characterize the responses of drought-resistant and drought-sensitive parental accessions under drought stress. By analyzing DEGs and DEPs within the BSA-identified intervals, five DEGs/DEPs with scores higher than five were identified as candidate drought resistance genes. These genes can facilitate the development of stress-tolerant crop varieties through targeted breeding or genetic engineering approaches.

## Figures and Tables

**Figure 1:**
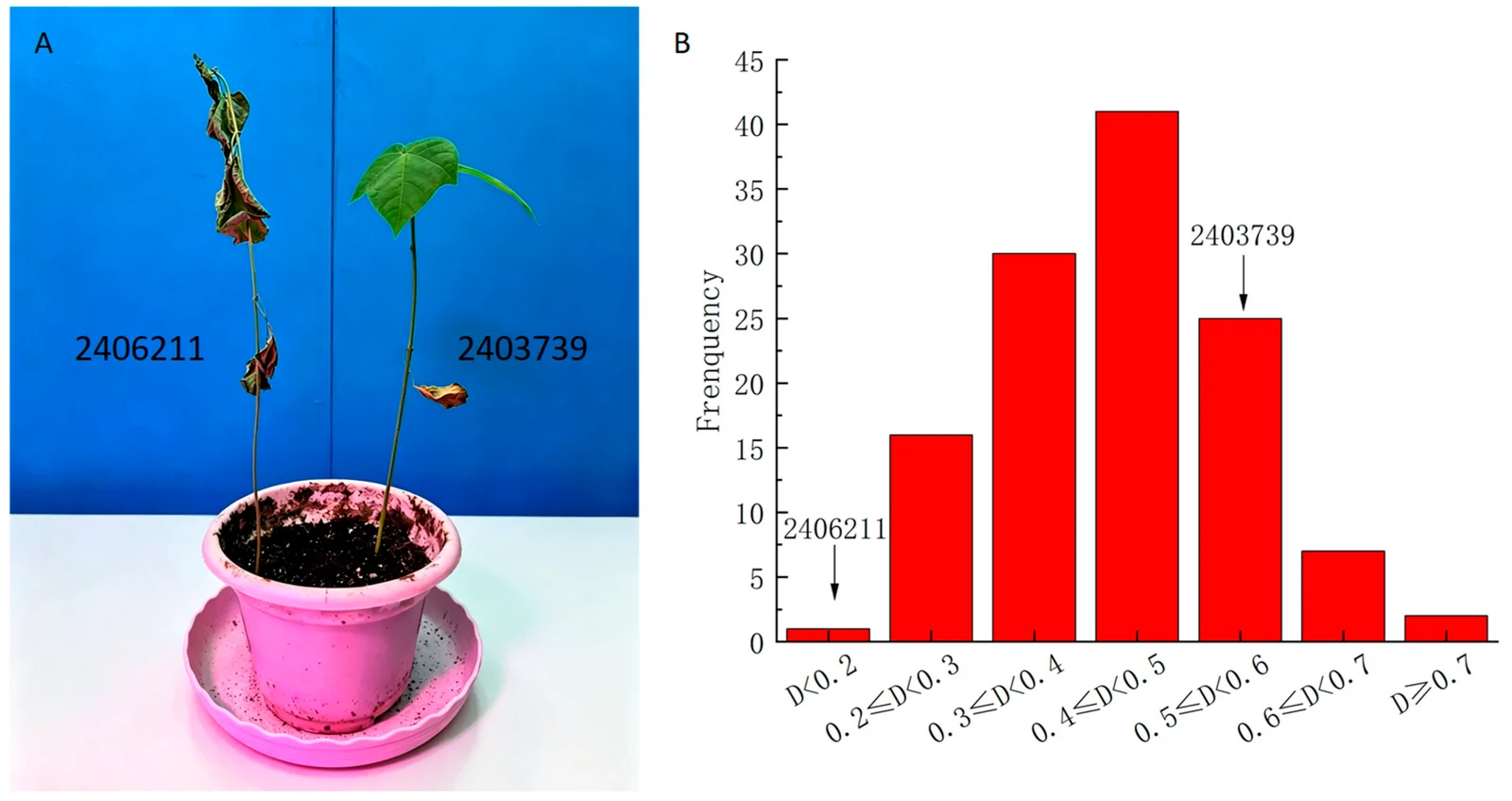
Drought resistance of cotton accessions 2403739, 2406211, and their F_2:3_ population. (**A**) Performance of the parental plants after rewatering following extreme drought stress, with the plant on the left being 2406211 (drought-sensitive) and the plant on the right being 2403739 (drought-resistant). (**B**) Frequency distribution of the comprehensive drought resistance measurement value (D value) in the F_2:3_ population. The larger the D value, the stronger the drought resistance. The frequency represents the number of offspring within a specific D value range.

**Figure 2:**
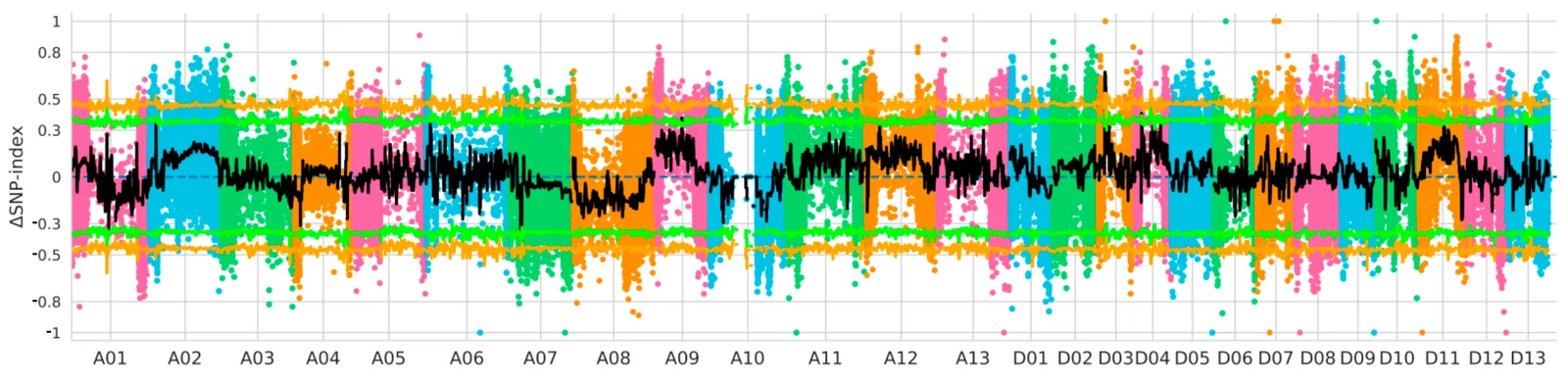
BSA-seq-based identification of five candidate quantitative trait loci (QTLs). The colored dots represent the calculated ΔSNP-index values. The black line indicates the average ΔSNP-index for all SNP loci within the sliding window, calculated using a sliding window approach. The green and orange lines represent the upper and lower boundaries of the 95% and 99% confidence intervals, respectively.

**Figure 3:**
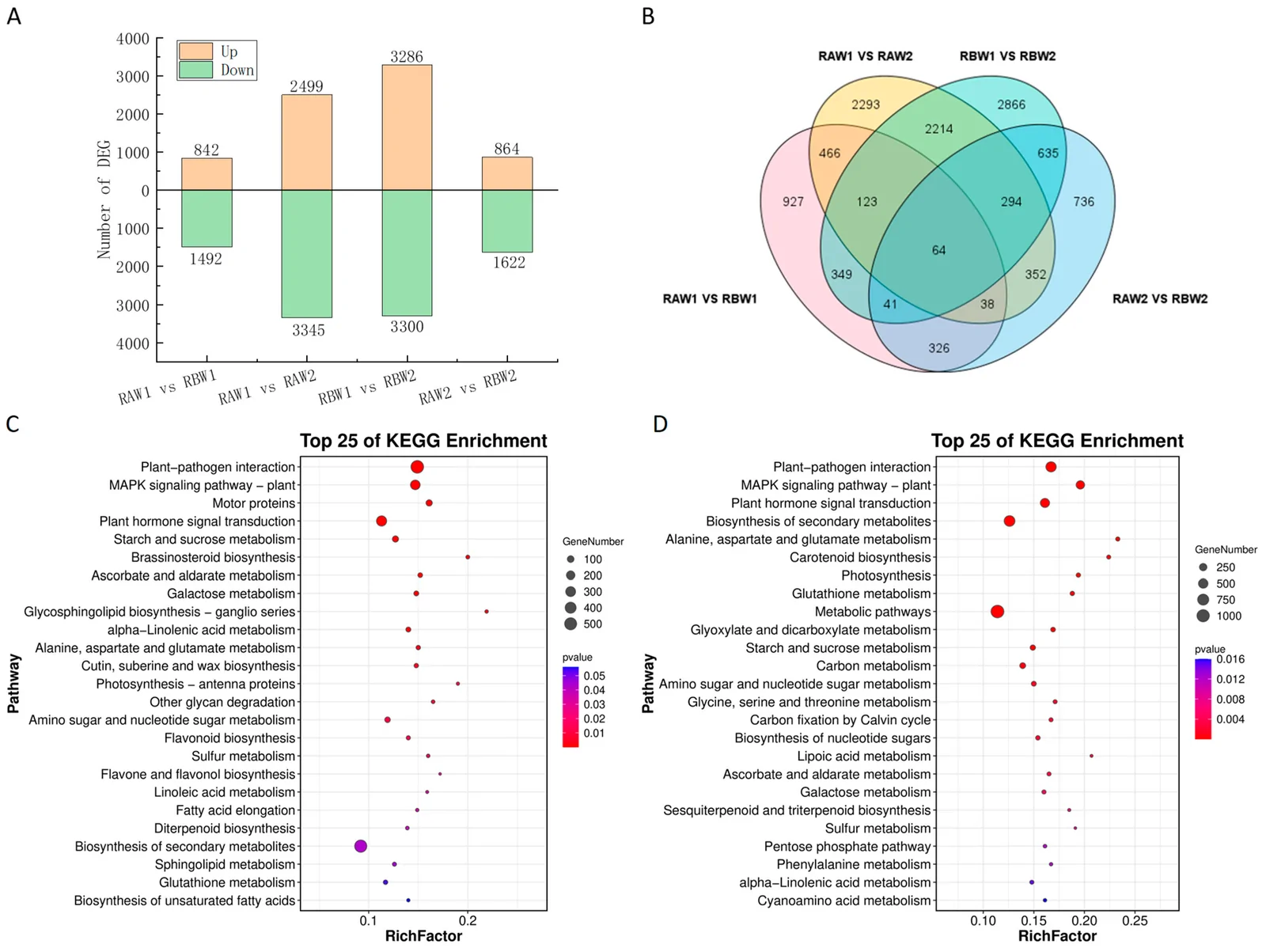
Analysis of DEGs identified in accession 2406211 (**A**) and accession 2403739 (**B**). A, Number of DEGs. B, Venn diagram of DEGs. (**C**,**D**) Bubble plot of KEGG enrichment analysis of DEGs for RAW1 versus RAW2 and RBW1 versus RBW2, respectively. RAW1, RNA-seq of accession A under control environment. RAW2, RNA-seq of accession A under drought stress. RBW1, RNA-seq of accession B under control environment. RBW2, RNA-seq of accession B under drought stress.

**Figure 4:**
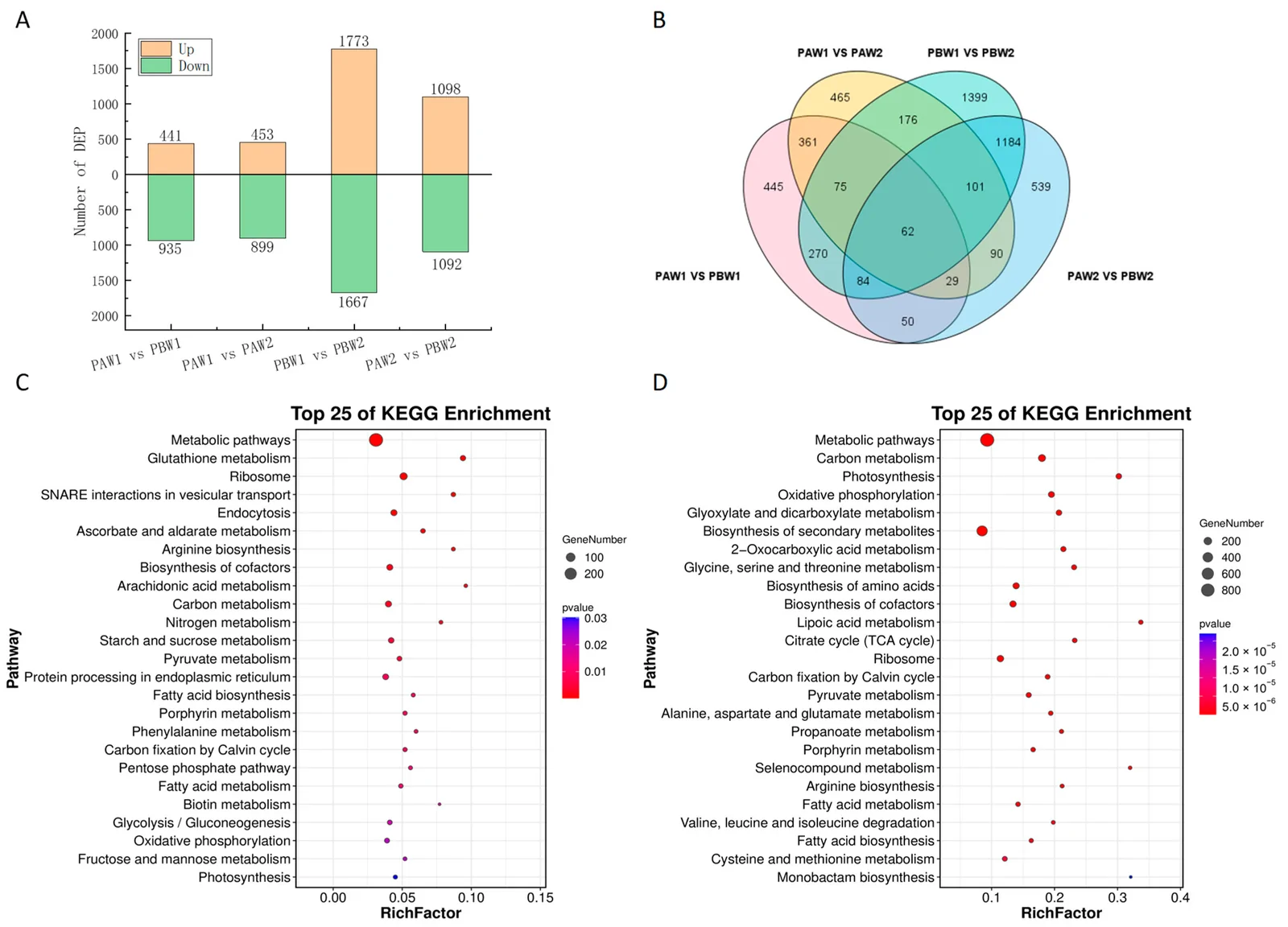
Analysis of DEPs identified in samples accession 2406211 (**A**) and accession 2403739 (**B**). A, Number of DEPs. B, Venn diagram of DEPs. (**C**,**D**) Bubble plot of KEGG enrichment analysis of DEPs comparing PAW1 versus PAW2 and PBW1 versus PBW2, respectively. PAW1, Proteome of accession A under control conditions; PAW2, Proteome of accession A under drought stress; PBW1, Proteome of accession B under control conditions; PBW2, Proteome of accession B under drought stress.

**Figure 5:**
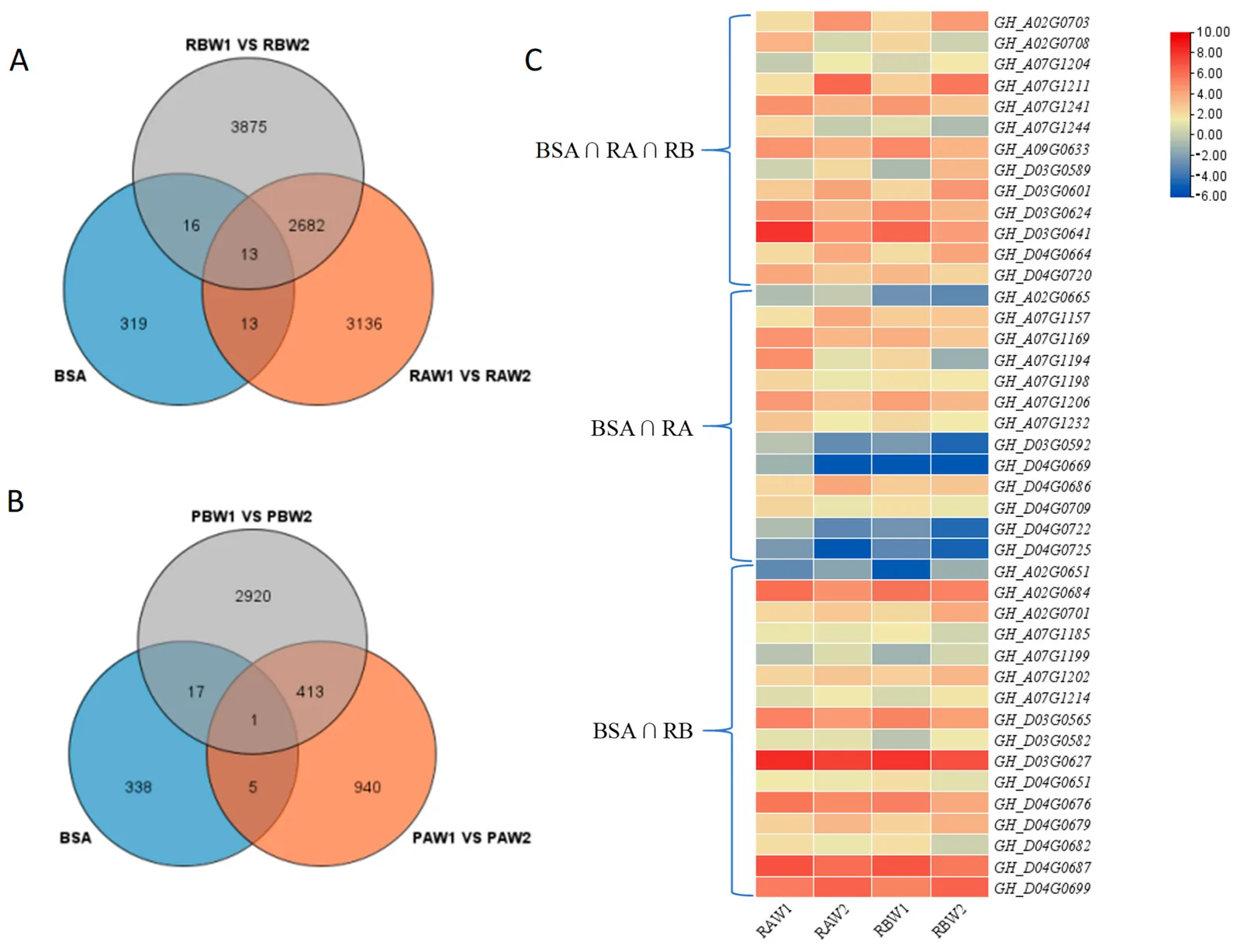
Multi-omics joint analysis for screening drought-resistant candidate genes in cotton. (**A**) Venn diagram of BSA, RAW1 versus RAW2, and RBW1 versus RBW2. (**B**) Venn diagram of BSA, PAW1 versus PAW2, and PBW1 versus PBW2. (**C**) Heatmap of differentially expressed genes (DEGs) showing log_2_ FPKM ratios from RNA-seq data within the BSA target intervals. “∩”, Intersection; PA, PAW1 versus PAW2; PB, PBW1 versus PBW2; PAW1, Proteome of accession A under control conditions; PAW2, Proteome of accession A under drought stress; PBW1, Proteome of accession B under control conditions; PBW2, Proteome of accession B under drought stress.

**Table 1:** Overview of sequencing data and alignment results of BSA-seq.

Bulk	Clean Reads	Data Generated (Gb)	Q30 (%)	Coverage 10× (%)	Depth (x)	GC (%)	Total Mapped Efficiency (%)	Perfect Mapped Efficiency (%)
2406211	4.68 × 10^8^	69.76	97.71	93.53%	28.59	37.35	98.08%	95.52
2403739	5.08 × 10^8^	75.64	97.87	93.00%	30.65	37.64	97.41%	95.60
sensitive-pool	4.07 × 10^8^	60.62	97.66	93.21%	25.82	36.29	99.79%	96.41
resistance-pool	3.89 × 10^8^	57.94	97.83	92.95%	24.68	36.21	99.77%	96.43

Q30 is the indicator representing the proportion of bases with a quality value greater than or equal to 30. Genome Coverage 10× refers to the ratio of the total number of bases obtained from sequencing to the size of the genome, indicating that the genome was covered at least 10 times.

**Table 2:** Estimates of the positions of the five identified QTLs.

QTL	Chromosome_ID	Chromosome Location (bp)		Size (Mb)
		Start	End	
*qD-A02-1*	A02	9,100,001	11,100,000	2
*qD-A07-1*	A07	18,200,001	20,700,000	2.5
*qD-A09-1*	A09	39,800,001	42,400,000	2.6
*qD-D03-1*	D03	8,900,001	13,800,000	4.8
*qD-D04-1*	D04	10,800,001	13,400,000	2.6
Total	—	—	—	14.5

**Table 3:** Drought-resistant candidate proteins identified through combined BAS-seq and proteomic analysis.

Category	ID	PAW1	PAW2	PBW1	PBW2
BSA ∩ PA ∩ PB	GH_D04G0702	188.06	0.15	109.50	238.54
BSA ∩ PA	GH_A02G0680	460.56	257.11	350.25	471.62
	GH_A07G1223	9799.51	5789.23	4962.32	7382.90
	GH_A07G1244	99.83	0.15	26.65	52.32
	GH_A09G0629	329.08	177.03	212.31	182.20
	GH_D03G0583	310.72	480.88	360.10	422.48
BSA ∩ PB	GH_A02G0687	597.68	314.00	519.60	283.46
	GH_A02G0689	384.23	452.80	237.02	555.65
	GH_A02G0691	127.66	115.09	197.98	39.05
	GH_A02G0712	448.06	259.01	252.69	444.37
	GH_A07G1169	1354.30	623.22	826.00	501.31
	GH_A07G1191	12,981.93	9895.37	14,133.08	32,259.45
	GH_A07G1192	701.84	746.47	617.02	1357.44
	GH_A07G1202	870.63	798.56	1341.97	750.08
	GH_A07G1210	619.31	687.13	953.38	610.42
	GH_D03G0572	2442.39	1654.82	989.66	1906.60
	GH_D03G0586	519.65	486.73	877.14	343.87
	GH_D03G0598	1544.67	2149.00	1340.56	2128.61
	GH_D04G0678	752.64	962.16	741.31	1566.85
	GH_D04G0687	5948.34	6376.65	7352.67	10,637.16
	GH_D04G0694	397.38	334.42	528.80	196.96
	GH_D04G0700	10,391.19	7159.48	8669.78	4707.31
	GH_D04G0719	7809.15	7200.49	6832.17	9410.45

“∩“, Intersection; PA, PAW1 versus PAW2; PB, PBW1 versus PBW2; PAW1, Proteome of accession A under control conditions; PAW2, Proteome of accession A under drought stress; PBW1, Proteome of accession B under control conditions; PBW2, Proteome of accession B under drought stress.

## Data Availability

The data presented in the study are deposited in Figshare DOI: 10.6084/m9.figshare.33413737.

## Supplementary Materials

The following supporting information can be downloaded at: https://www.mdpi.com/article/10.3390/biology15201902/s1, Figure S1: Pearson correlation analysis of RNA-seq results of accession 2406211 (A) and accession 2403739 (B); Figure S2: PCA of RNA-seq results of accession 2406211 (A) and accession 2403739 (B); Figure S3: Rich factor of GO enrichment analysis of DEGs for RAW1 versus RAW2 and RBW1 versus RBW2, respectively. R, RNA-seq; A, accession 2406211; B, accession 2403739, W1, control condition, W2, drought stress; Figure S4: Pearson correlation analysis of proteomic results of accession 2406211 (A) and accession 2403739 (B); Figure S5: PCA analysis of proteomic results of accession 2406211 (A) and accession 2403739 (B); Figure S6: Rich factor of GO enrichment analysis of DEPs for PAW1 versus PAW2 and PBW1 versus PBW2, respectively. P, proteome; A, accession 2406211; B, accession 2403739, W1, control condition, W2, drought stress; Table S1:The densities of SNP and short InDel markers across different chromosomes; Table S2: The sequencing quality of RNA-seq data; Table S3: The sequence alignment of RNA-seq data; Table S4: The detailed information on the KEGG enrichment results of RAW1 versus RAW2; Table S5: The detailed information on the KEGG enrichment results of RBW1 versus RBW2; Table S6: The Pearson correlation coefficients among the three replicates of each sample; Table S7: The detailed information on the KEGG enrichment significant pathway of PAW1 versus PAW2; Table S8: The detailed information on the KEGG enrichment significant pathway of PBW1 versus PBW2; Table S9: Annotation of drought-resistant candidate genes identified through joint analysis of QTL and RNA-seq; Table S10: Annotation of drought-resistant candidate genes identified through joint analysis of BSA and proteomics; Table S11: Common genes identified through the joint analysis of BSA with transcriptome data and BSA with proteome data; Table S12: Exonic and upstream variant in candidate genes; Table S13: The DEG scores within the QTL interval; Table S14: Commonly differentially expressed genes identified in transcriptome and proteome analyses.
