# Supplementary material for: Bulk Segregant Analysis Integrated with Transcriptomic and Proteomic Approaches Reveals Candidate Genes Associated with Drought Tolerance in Cotton
Source: Biology (Basel). 2026 Oct 21;15(20):1902. doi: 10.3390/biology15201902 (PMC13701935; doi:10.3390/biology15201902)
Supplement: Supplementary file 1 [file biology-15-01902-s001.zip › Supplement-Figure.pdf]

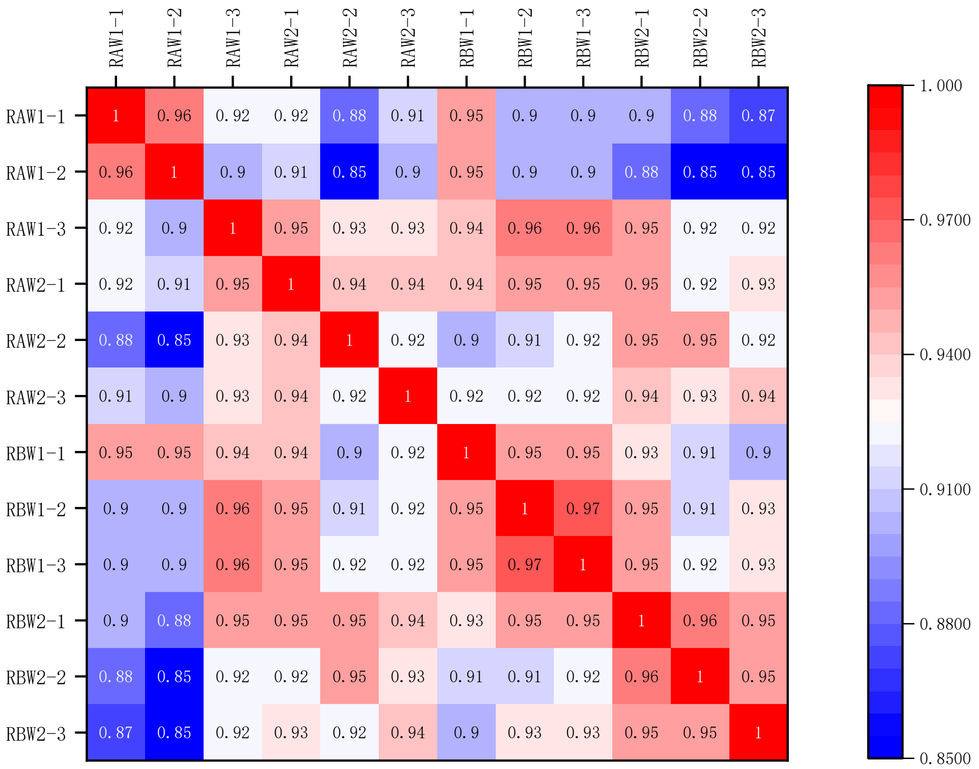

Figure S1. Pearson correlation analysis of RNA-seq results of accession 2406211 (A) and accession 2403739 (B).

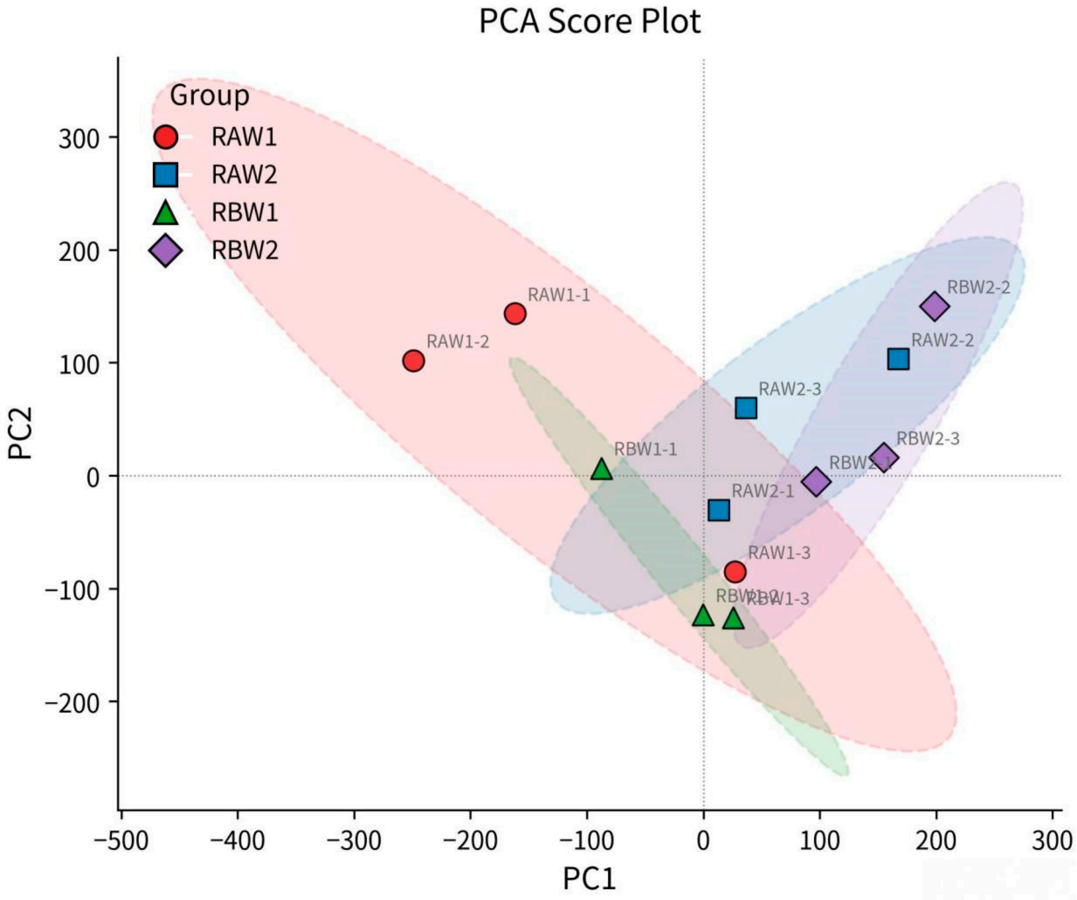

Figure S2. PCA of RNA-seq results of accession 2406211 (A) and accession 2403739 (B).

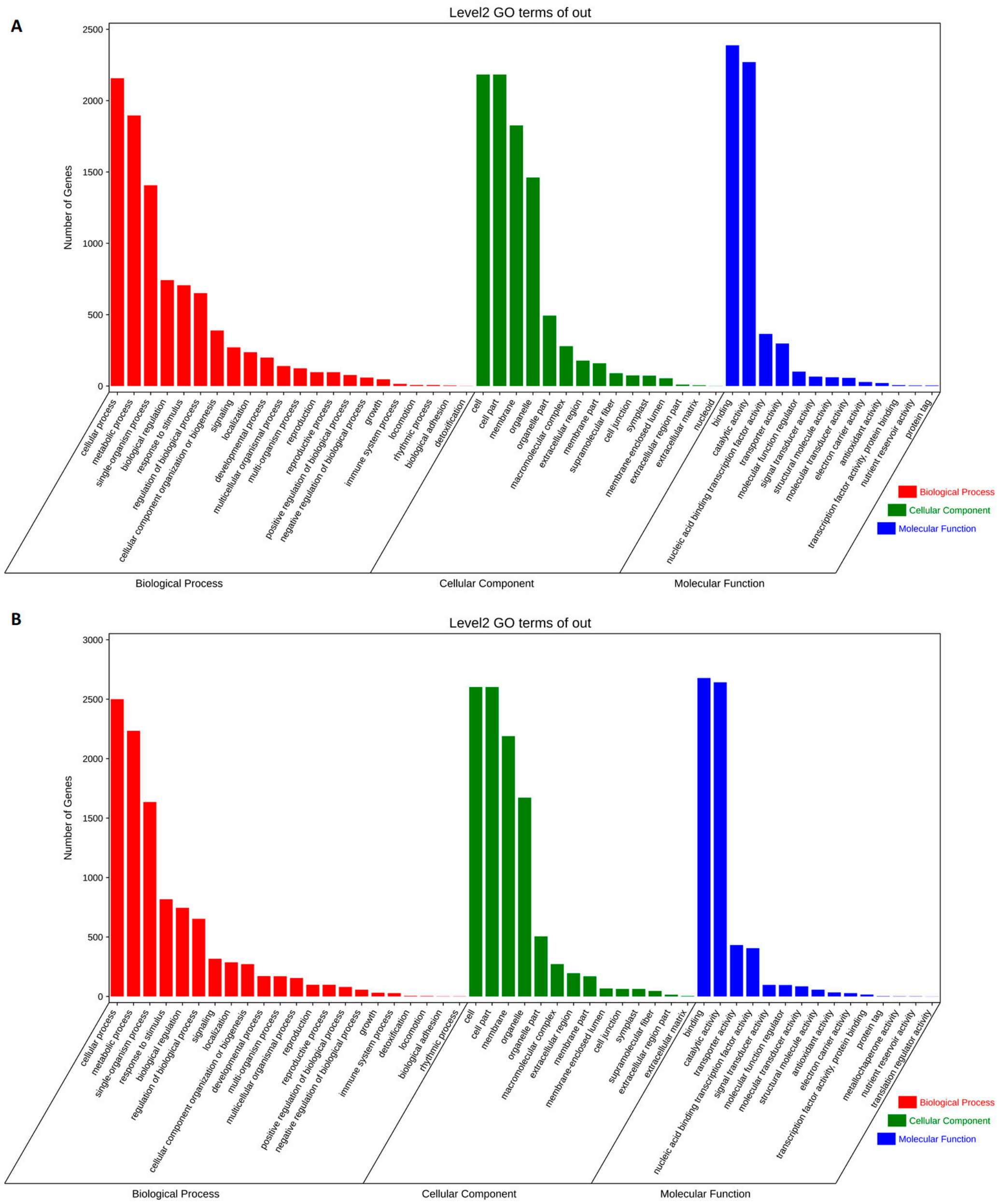

Figure S3. Rich factor of GO enrichment analysis of DEGs for RAW1 versus RAW2 and RBW1 versus RBW2, respectively. R, RNA-seq; A, accession 2406211; B, accession 2403739, W1, control condition, W2, drought stress.

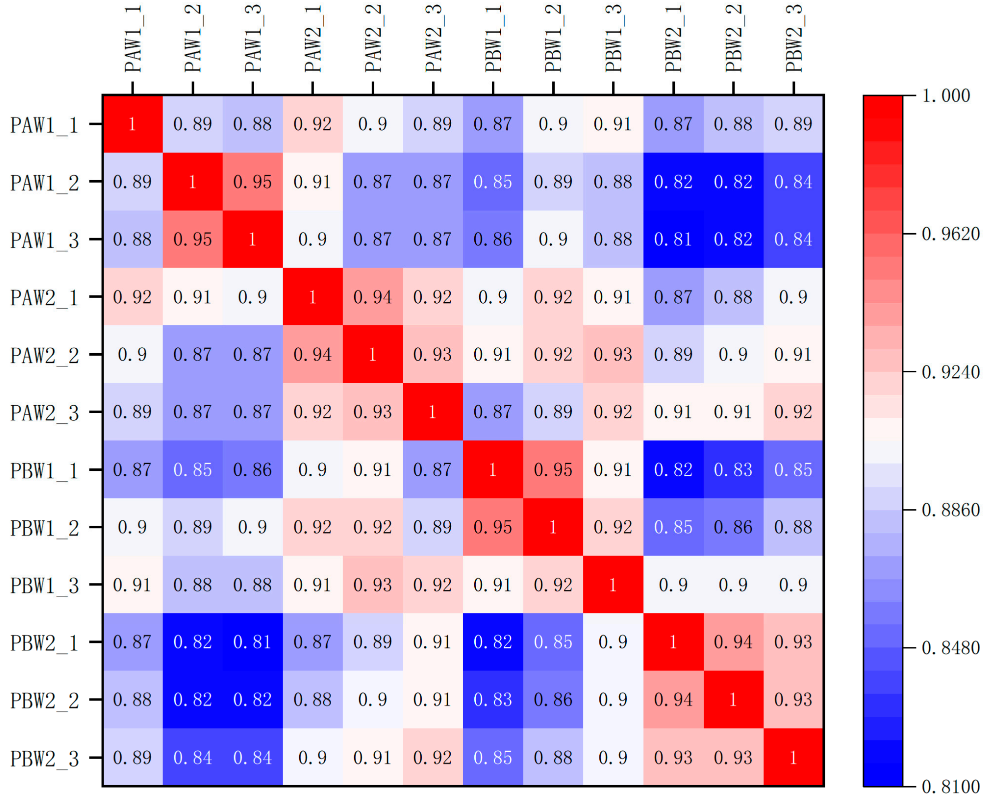

Figure S4. Pearson correlation analysis of proteomic results of accession 2406211 (A) and accession 2403739 (B).

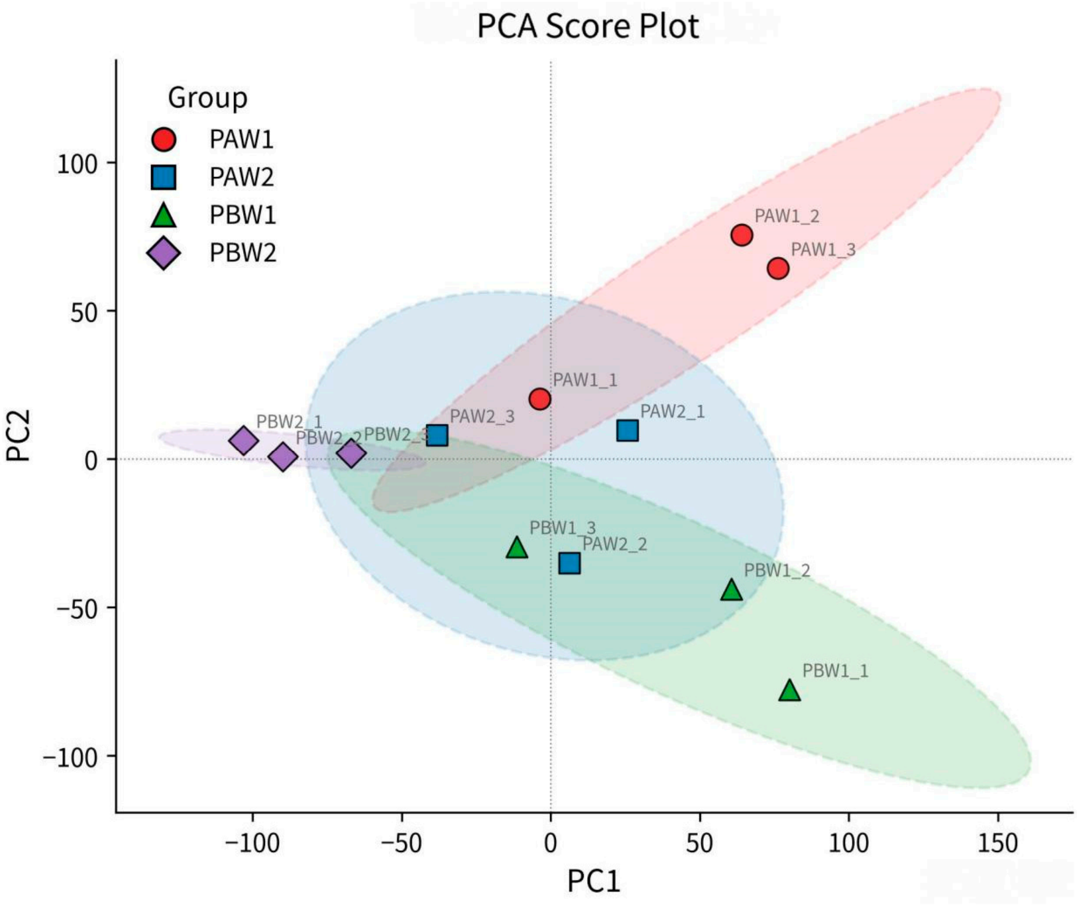

Figure S5. PCA analysis of proteomic results of accession 2406211 (A) and accession 2403739(B).

A

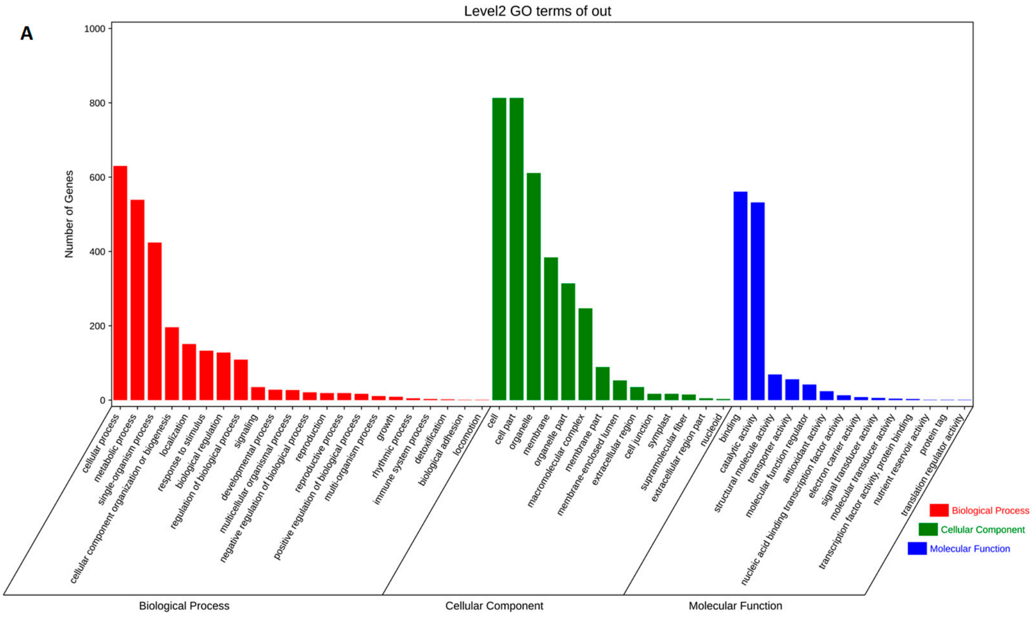

B

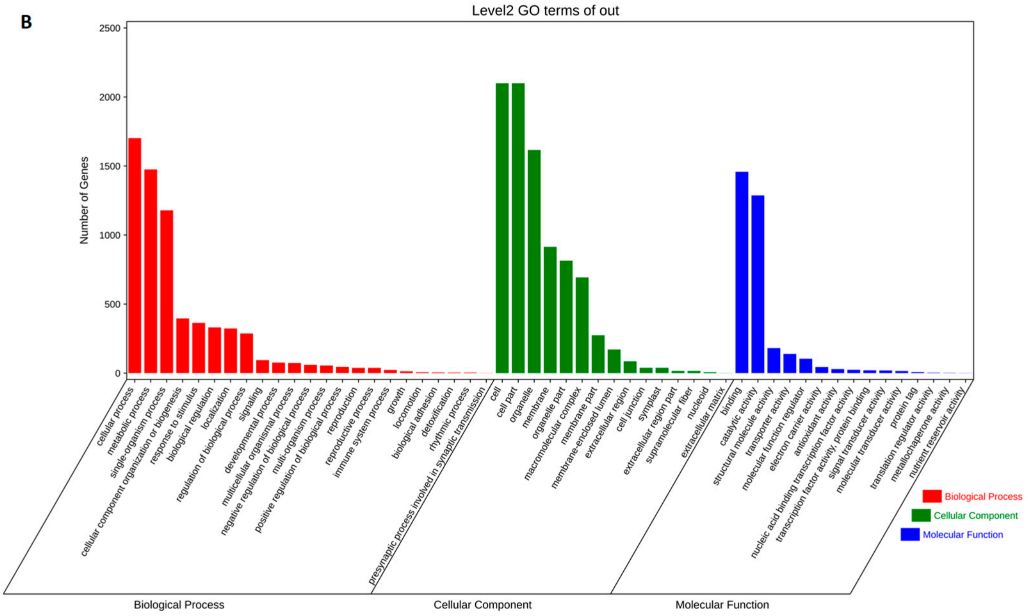

Figure S6. Rich factor of GO enrichment analysis of DEPs for PAW1 versus PAW2 and PBW1 versus PBW2, respectively. P, proteome; A, accession 2406211; B, accession 2403739, W1, control condition, W2, drought stress.
